# Supplementary material for: In vivo noninvasive systemic myography of acute systemic vasoactivity in female pregnant mice
Source: Nat Commun. 2023 Oct 9;14:6286. doi: 10.1038/s41467-023-42041-8 (PMC10562381; doi:10.1038/s41467-023-42041-8)
Supplement: Supplementary file 1 — Supplementary Information [file 41467_2023_42041_MOESM1_ESM.pdf]

In vivo noninvasive systemic myography of acute systemic vasoactivity in female pregnant mice

S.1. Classifying Vasodilation by Artery

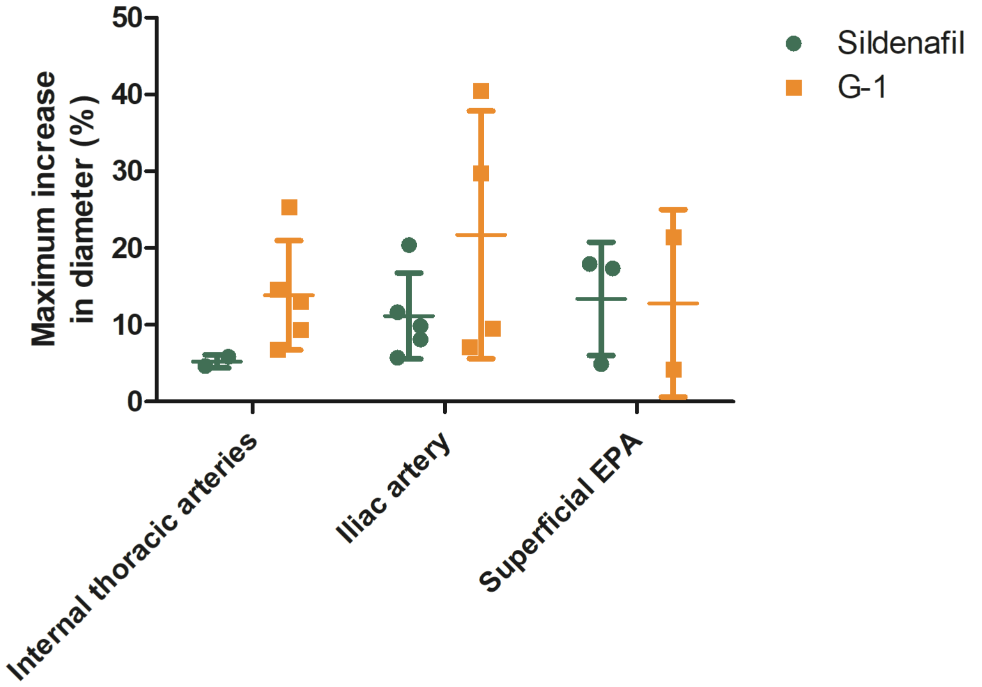

Fig.S.1. Individual arterial responses. Scatter plot of acute vasodilation of individual arteries due to sildenafil and G-1 with mean and standard deviation (n= 6 mice/group).

S.2. Assessing the accuracy of vasodilation measurement

To assess the metrics of photoacoustic tomography in measuring vessel diameter, we compared the extent of dilation of a tube phantom using camera video images and photoacoustic images. We used a silicone tube (1/32” ID, 1/16” OD) filled with indocyanine green (ICG) dye to mimic a vessel. First, we applied axial tension on a small section of the tube manually to make it easily inflatable. Then, we knotted one side of the tube to secure the pressure. We used a syringe pump to apply pressure at a flow rate of 0.9 mL/min for 300 µL using infuse flow direction. We imaged the tube phantom with the TriTom system before

inflation and with a condition of 8s stable / 20s inflation / 8s stable at an inflated state. We also recorded video during photoacoustic imaging using TriTom built-in camera.

We reconstructed the photoacoustic images using the reconstruction software. Then we manually identified the 2D cross-section image of the tube along the y-z plane from the photoacoustic image volume. Using MATLAB, we measured the diameter of the tube from pre- and post-inflation photoacoustic images. In addition, we measured the diameter of the tube from the camera video image before inflation and post-inflation. The increase in the diameter of the tube was calculated using equation (3) for both imaging. To compare the dilation measured from photoacoustic images with the dilation measured we calculated the difference between the measured increase in diameter.

Fig. S.2a and b show the tube phantom image for both systems. We found that the extent of dilation measured using a camera image is greater (~40%) than the extent of dilation measured using a photoacoustic image (Fig. S.2c). This difference matches our in vivo vasodilation measurement difference for B-mode and photoacoustic imaging (Fig. 2e).

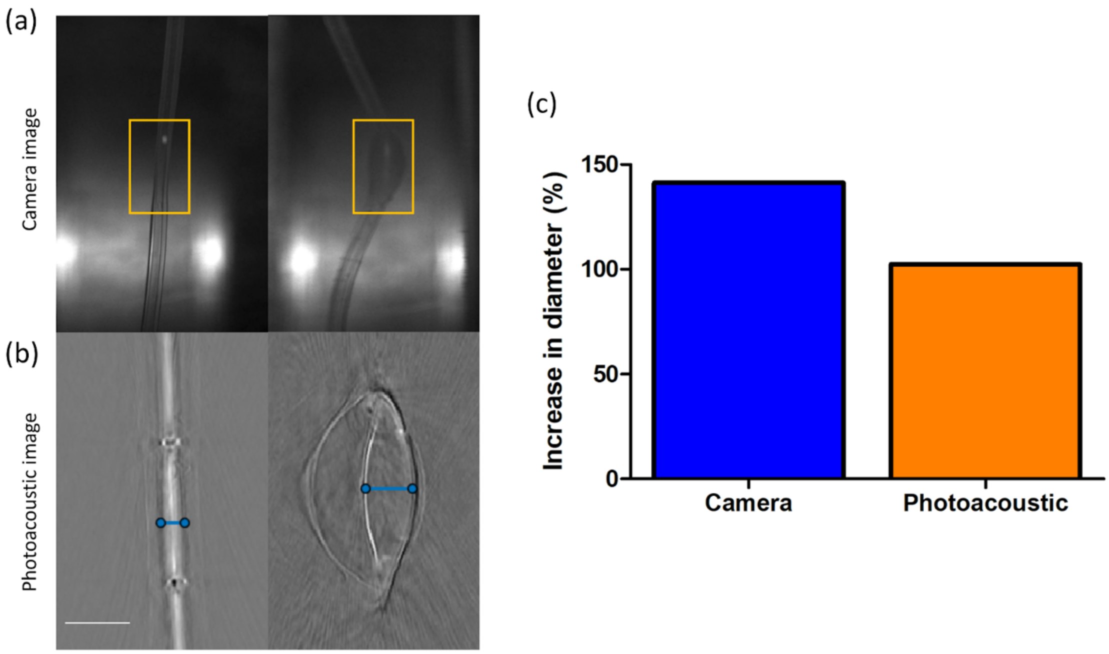

Fig. S.2. Validation of measured diameters using a tube phantom. (a) Camera image of tube phantom before and post dilation. Yellow box is showing the section where the tube inflated from 1.5875 mm to 4.24 mm. (b) Photoacoustic image of tube phantom filled with ICG dye before (1.7 mm) and post dilation (3.8 mm) at 778 nm wavelength. Scale bar in 5 mm. (c) Bar graph of increase in diameter measured from camera image and photoacoustic image. Camera image shows ~43% greater increase in diameter than photoacoustic image.

S.3. Effect of vasoconstrictor on maternal vasculature

We treated a CD-1 pregnant mouse with endothelin-1 (E7764, Sigma-Aldrich) to observe the effect of a vasoconstrictor on arteries. A volume of 0.1 mL endothelin-1 (0.032 µg/kg of body weight in PBS) was administered through the right jugular vein catheter, followed by a flush of 0.1 mL PBS, while imaging continuously. The photoacoustic images before and post-injection were acquired at 1064 nm wavelength.

Using the same methods as developed to assess vasodilation, we calculated that the system arteries (specifically the internal thoracic and iliac arteries) minimally decreased in diameter (2.3%). In Figure S.3. the green arrow is showing the decrease in the diameter of the iliac artery. It is likely we are not able to fully capture all vasoconstricting vessels, since they may reduce to diameters smaller than the spatial resolution of the imaging system.

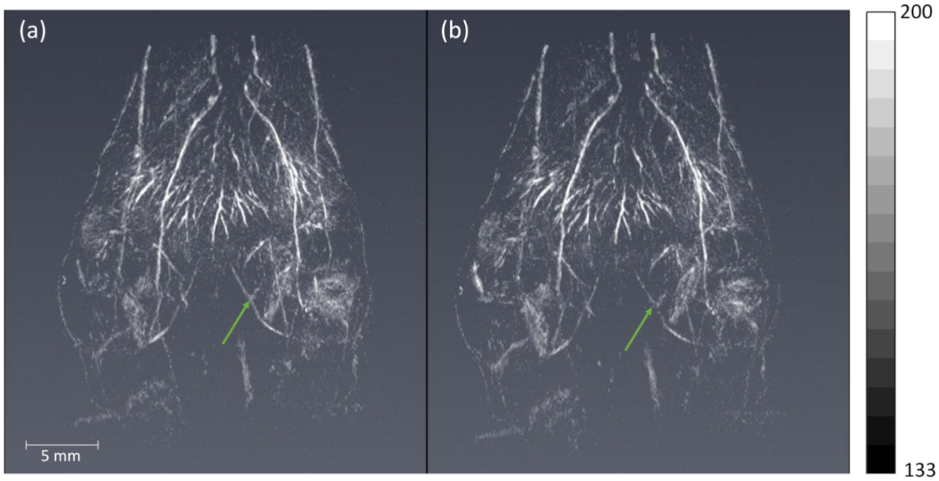

Fig. S.3. Representative image of vasoconstriction in response to endothelin-1. (a) Pre-injection at 1064 nm (b) 1.25 minutes post-injection.

S.4. In vivo photoacoustic imaging of placental oxygenation

Changes in placental oxygenation were monitored post-injection for PBS (n=5) and sildenafil (n=5). The animals were prepared for surgery and imaged using the protocol described above. For spectral photoacoustic imaging, we acquired images at wavelengths of 690, 808 and 850 nm. Hemoglobin and oxyhemoglobin have relative peak optical absorption at 690 and 850 nm whereas, 808 nm is the isosbestic point of optical absorption for both.

TriTom system was used for 3D volumetric image acquisition. The animal was imaged at 690, 808 and 850 nm before drug administration. The animal was imaged spectrally (690 nm, 808 nm, 850 nm) post injection for 30 minutes. For placental oxygenation measurement, the acquired images were reconstructed and normalized using the method described above. We manually segmented the 3D volumes of placenta from reconstructed 3D images using MATLAB. We estimated the concentrations of Hb and HbO_2 for the whole 3D volume using a linear least-squares spectral unmixing algorithm [1,2]. From the estimated concentration of Hb and HbO_2, the relative sPO_2 was calculated following Eq. (1):

$$Relative\ sPO_2 = \frac{[HbO_2]}{[Hb] + [HbO_2]} \times 100\% \quad (3)$$

The pseudo color display of the relative sPO_2 of each pixel was implemented following our previously developed methods [3].

Our experiment on placental oxygenation measurement in response to sildenafil showed a slight increase in placental oxygenation over time. We compared the maximum increase in placental oxygenation at 30 minutes post injection in response to PBS and sildenafil (Fig S.4b). We did not any find significant differences between two groups.

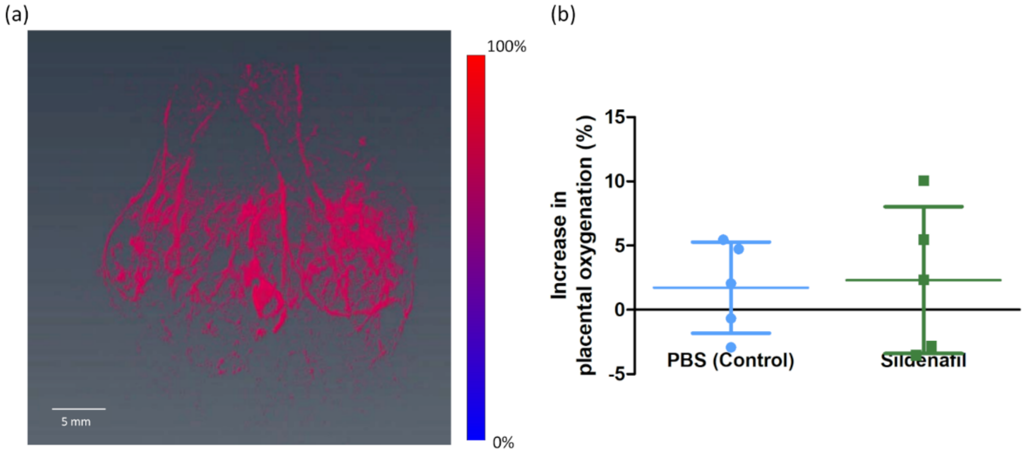

Fig. S.4. Placental oxygenation after sildenafil injection. (a) Representative image of whole-body oxygenation using a pseudo red and blue colormap. We used a linear colormap where blue is deoxygenated, and red is oxygenated hemoglobin. (b) Scatter plot of maximum increase in placental oxygenation in response to PBS and sildenafil with mean and standard deviation (n=5 mice/group).

Supplementary References

1. Luke, G. P. & Emelianov, S. Y. Label-free Detection of Lymph Node Metastases with US-guided Functional Photoacoustic Imaging. *Radiology* **277**, 435-442 (2015).
2. Bayer, C. L., Wlodarczyk, B. J., Finnell, R. H. & Emelianov, S. Y. Ultrasound-guided spectral photoacoustic imaging of hemoglobin oxygenation during development. *Biomed Opt Express* **8**, 757-763 (2017).
3. Huda, K., Wu, C., Sider, J. G. & Bayer, C. L. Spherical-view photoacoustic tomography for monitoring in vivo placental function. *Photoacoustics* **20**, 100209 (2020).
