# Supplementary material for: In vivo noninvasive systemic myography of acute systemic vasoactivity in female pregnant mice
Source: Nat Commun. 2023 Oct 9;14:6286. doi: 10.1038/s41467-023-42041-8 (PMC10562381; doi:10.1038/s41467-023-42041-8)
Supplement: Supplementary file 3 — Reporting Summary [file 41467_2023_42041_MOESM3_ESM.pdf]

nature portfolio

Corresponding author(s): Carolyn L. Bayer

Last updated by author(s): Sep 11, 2023

Reporting Summary

Nature Portfolio wishes to improve the reproducibility of the work that we publish. This form provides structure for consistency and transparency in reporting. For further information on Nature Portfolio policies, see our Editorial Policies and the Editorial Policy Checklist.

Statistics

For all statistical analyses, confirm that the following items are present in the figure legend, table legend, main text, or Methods section.

n/a	Confirmed	
☐	☒	The exact sample size (*n*) for each experimental group/condition, given as a discrete number and unit of measurement
☐	☒	A statement on whether measurements were taken from distinct samples or whether the same sample was measured repeatedly
☐	☒	The statistical test(s) used AND whether they are one- or two-sided<br>*Only common tests should be described solely by name; describe more complex techniques in the Methods section.*
☐	☒	A description of all covariates tested
☐	☒	A description of any assumptions or corrections, such as tests of normality and adjustment for multiple comparisons
☐	☒	A full description of the statistical parameters including central tendency (e.g. means) or other basic estimates (e.g. regression coefficient) AND variation (e.g. standard deviation) or associated estimates of uncertainty (e.g. confidence intervals)
☐	☒	For null hypothesis testing, the test statistic (e.g. *F*, *t*, *r*) with confidence intervals, effect sizes, degrees of freedom and *P* value noted<br>*Give P values as exact values whenever suitable.*
☒	☐	For Bayesian analysis, information on the choice of priors and Markov chain Monte Carlo settings
☒	☐	For hierarchical and complex designs, identification of the appropriate level for tests and full reporting of outcomes
☒	☐	Estimates of effect sizes (e.g. Cohen's *d*, Pearson's *r*), indicating how they were calculated

Our web collection on statistics for biologists contains articles on many of the points above.

Software and code

Policy information about availability of computer code

Data collection	The PhotoSound Technologies prototype Tritom system (no version available) was used to collect all data.
Data analysis	PhotoSound Image Reconstruction V3.0.3, GraphPad Prism 5.0, G*Power 3.1.9.6 for Mac, Amira V6.0.1, and Matlab 2021b were used to process all data. The post-reconstruction image processing codes are available upon request; this code includes implementation of the Frangi vesselness filter and linear fitting of the multiwavelength photoacoustic data to the oxygen saturation of hemoglobin, both of which are previously published and documented methods.

For manuscripts utilizing custom algorithms or software that are central to the research but not yet described in published literature, software must be made available to editors and reviewers. We strongly encourage code deposition in a community repository (e.g. GitHub). See the Nature Portfolio guidelines for submitting code & software for further information.

Data

Policy information about availability of data

All manuscripts must include a data availability statement. This statement should provide the following information, where applicable:

- Accession codes, unique identifiers, or web links for publicly available datasets
- A description of any restrictions on data availability
- For clinical datasets or third party data, please ensure that the statement adheres to our policy

Source data file is provided as Source Data.xlsx as a supplementary. The image dataset is uploaded on Dryad (https://doi.org/10.5061/dryad.sn02v6x9n)

Research involving human participants, their data, or biological material

Policy information about studies with human participants or human data. See also policy information about sex, gender (identity/presentation), and sexual orientation and race, ethnicity and racism.

Reporting on sex and gender	N/A
Reporting on race, ethnicity, or other socially relevant groupings	N/A
Population characteristics	N/A
Recruitment	N/A
Ethics oversight	N/A

Note that full information on the approval of the study protocol must also be provided in the manuscript.

Field-specific reporting

Please select the one below that is the best fit for your research. If you are not sure, read the appropriate sections before making your selection.

☒ Life sciences ☐ Behavioural & social sciences ☐ Ecological, evolutionary & environmental sciences

For a reference copy of the document with all sections, see nature.com/documents/nr-reporting-summary-flat.pdf

Life sciences study design

All studies must disclose on these points even when the disclosure is negative.

Sample size	A priori power analysis was performed with a power of 0.80 and significance of 0.05 to determine the sample size using G*Power software (Heinrich-Heine-Universität, Dusseldorf, Germany).
Data exclusions	Animals with large motion artifacts due to irregular breathing were excluded from data analysis.
Replication	n=6 mice were used for assessment of systemic vasculature, and n=8 mice were used for assessment of response to vasodilators. Motion artifacts which prevented longitudinal tracking of the segmented vasculature was an exclusionary criteria from the final data set. The resulting number of qualified animals are reported in the manuscript and captions.
Randomization	The samples were randomly assigned to each cohort.
Blinding	The investigators were not blinded to group allocation during data collection and data analysis, as a single operator both collected and analyzed the data given the level of technical expertise required.

Reporting for specific materials, systems and methods

We require information from authors about some types of materials, experimental systems and methods used in many studies. Here, indicate whether each material, system or method listed is relevant to your study. If you are not sure if a list item applies to your research, read the appropriate section before selecting a response.

Materials & experimental systems

n/a	Involved in the study	
☒	☐	Antibodies
☒	☐	Eukaryotic cell lines
☒	☐	Palaeontology and archaeology
☐	☒	Animals and other organisms
☒	☐	Clinical data
☒	☐	Dual use research of concern
☒	☐	Plants

Methods

n/a	Involved in the study	
☒	☐	ChIP-seq
☒	☐	Flow cytometry
☒	☐	MRI-based neuroimaging

Animals and other research organisms

Policy information about studies involving animals; ARRIVE guidelines recommended for reporting animal research, and Sex and Gender in Research

Laboratory animals	Timed pregnant CD-1 mice, 8-12 weeks of age were acquired from Charles River. Housing conditions for the mice, dark/light cycle, ambient temperature and humidity are reported in the manuscript.
Wild animals	No wild animals were used in the study.
Reporting on sex	All mice were female since we report studying vasodilation of systemic arteries and the placenta in pregnant mice.
Field-collected samples	No field-collected samples were used in the study.
Ethics oversight	Tulane University IACUC approved all reported studies.

Note that full information on the approval of the study protocol must also be provided in the manuscript.
